# Cognitive impairment in young adults with post COVID-19 syndrome

**DOI:** 10.1038/s41598-023-32939-0

**Published:** 2023-04-19

**Authors:** Elena Herrera, María del Carmen Pérez-Sánchez, Romina San Miguel-Abella, Arrate Barrenechea, Claudia Blanco, Lucía Solares, Lucía González, Clara Iza, Isabel Castro, Elena Nicolás, Damián Sierra, Paula Suárez, María González-Nosti

**Affiliations:** Department of Psychology, University of Oviedo, Oviedo, Spain

**Keywords:** Cognitive neuroscience, Psychology

## Abstract

In this study, we aimed to examine different cognitive domains in a large sample of patients with post COVID-19 syndrome. Two hundred and fourteen patients, 85.04% women, ranged 26 to 64 years (mean = 47.48 years) took part in this investigation. Patients’ processing speed, attention, executive functions and various language modalities were examined online using a comprehensive task protocol designed for this research. Alteration in some of the tasks was observed in 85% of the participants, being the attention and executive functions tests the ones that show the highest percentage of patients with severe impairment. Positive correlations were observed between the age of the participants in almost all the tasks assessed, implying better performance and milder impairment with increasing age. In the comparisons of patients according to age, the oldest patients were found to maintain their cognitive functions relatively preserved, with only a mild impairment in attention and speed processing, while the youngest showed the most marked and heterogeneous cognitive impairment. These results confirm the subjective complaints in patients with post COVID-19 syndrome and, thanks to the large sample size, allow us to observe the effect of patient age on performance, an effect never reported before in patients with these characteristics.

## Introduction

Ten to fifteen percent of the patients who have passed COVID-19 report that they continue to experience a wide range of symptoms even several months after the infection. This disorder, named “post COVID-19 syndrome”, affects mainly women aged between 40 and 55 years, most of whom have undergone mild or even asymptomatic COVID-19^1–3^. Headache, fatigue, respiratory difficulties and cognitive dysfunction are some of the most common symptoms of the more than 200 reported by the patients. In 2021, this syndrome was defined by the World Health Organization^4^ as a condition that “occurs in individuals with a history of probable or confirmed SARS-CoV-2 infection, usually 3 months from the onset of COVID-19 with symptoms that last for at least 2 months and cannot be explained by an alternative diagnosis”.

Regarding the underlying causes of this syndrome, three hypotheses are already being considered: One of them is the persistence of the virus in the organism. Thus, some studies have demonstrated the presence of the virus in the digestive tract^5^ and in the olfactory mucosa^6^, from which it might penetrate the central nervous system. The second theory concerns the so-called cytokine storm triggered by the virus in its acute phase^7^. This is a pathological reaction of the patient’s immune system that causes inflammation associated with the severity of the disease and the persistence of symptoms. According to a recent study^8^ many infected patients have antibodies specific for the ACE2 enzyme. Binding of SARS-CoV-2 to ACE2 results in cytokine storming and increased inflammation during SARS-CoV-2 infection. The third hypothesis regards the possible existence of autoantibodies in COVID-19 that may act against immunomodulatory proteins, perturbing immune function^9^.

Regardless of the causes, this new syndrome significantly interferes with patients’ activities and cognitive symptoms are usually those that cause them the most concern.

Bertuccelli et al.^10^ recently conducted a comprehensive review of studies on cognitive impairment in infected individuals. The review yields interesting results: the cognitive domain reported as impaired in the largest number of studies (8/16) are executive functions. Seven of the 12 studies that evaluated attention found significant impairment in all types of attention with higher error rates and slower reaction times. Memory was evaluated in ten studies, mainly verbal and working memory. Only one of the studies found alterations in immediate recall, while two of them reported impaired delayed recall. As for working memory, significant alterations were found in only two out of seven studies.

Regarding language, only one study included specific tasks to assess this cognitive domain and 97% of the patients obtained normal scores (using the Boston Naming Test). Finally, visuospatial functions were evaluated through specific tasks in 5 studies, although no altered performance of the patients was found in any of them.

Some other studies not included in these reviews show similar results. In the study by Krishnan et al.^11^ with 20 patients, alterations in attention, processing speed and executive functions were found. On the other hand, in the study conducted by Delgado-Alonso et al.^12^ with 50 Spanish patients, impaired performance was found in the cognitive domains of attention, executive functions, episodic memory and visuospatial skills. Dressing et al.^13^, however, failed to find impairments despite the extensive neuropsychological battery they applied to their 31 patients.

Although there is no doubt that these studies are of enormous interest in trying to clarify the cognitive profile of patients with post COVID-19 syndrome, some limitations should be pointed out to treat the results with caution. The main one, in our opinion, is that most of the studies do not differentiate whether the sample includes only patients with post COVID-19 syndrome, or also patients with sequelae of COVID-19. The overall characteristics of the samples of the 25 studies included in the mentioned review show mean ages around 60 years, a majority of males (55%) and a high percentage of patients who required admission to the ICU, all of which suggests that a large proportion of the participants had post-COVID sequelae. This difference is not trivial, since the underlying causes of COVID-19 sequelae are organ damage from a severe form of the disease. In the case of post COVID-19 syndrome, however, no structural brain damage is observed on neuroimaging^13^. Although at the moment there is no evidence of structural damage in patients with this syndrome, metabolic differences are observed in comparison with healthy individuals. A recent study shows bilateral hypometabolism in the olfactory gyrus, the left temporal lobe (including the amygdala and the hippocampus) and the talamus. This metabolic pattern was associated with increased dysfunction and the presence of cognitive symptoms, pain, decreased olfaction and insomnia^14^.

When considering exclusively patients with post COVID-19 syndrome, a recently meta-analysis reported that 32% of patients experienced “brain fog”, 28% memory problems and 22% attentional difficulties. With respect to some sociodemographic characteristics of the patients, it is particularly noteworthy that when considering the total sample, 59% were women with a mean age of 52 years. However, when the non-hospitalized patients were analyzed, the percentage of women increased to 78% and the mean age decreased to 46 years^15^.

Therefore, everything points to the fact that the sequelae of COVID-19 and post COVID-19 syndrome are different disorders, with different causes and, it can be assumed, a different cognitive profile.

Thus, the small number of studies on this topic and the methodological heterogeneity, make it difficult to define a clear pattern of cognitive impairment. If we add to this the fact that the small number of participants included in the studies prevents comparisons between patients according to age or other characteristics, we find ourselves with a panorama of lack of consensus that worries patients and hinders the adoption of clear, evidence-based action protocols.

The purpose of this study, therefore, is to identify the cognitive features of a large sample of patients with a diagnosis of post COVID-19 syndrome (or compatible symptoms). For this goal, neuropsychological evaluations have been performed on individuals who, after having overcome the acute phase of COVID-19, report cognitive complaints lasting more than 4 months.

## Method

### Participants

The study was conducted with 214 patients with a diagnosis of COVID-19 and diagnosis or suspected post COVID-19 syndrome based on the approved WHO definition. Post-COVID syndrome was recognized when, after a period of 3 months after COVID-19, new symptoms that appeared during the acute phase remained and/or worsened. The mean age of the participants was 47.48 years (SD = 7.35), with ages between 26 and 64. Among the total sample, 182 (85.04%) were women (see Table 1).

**Table 1:** Demographic and clinical characteristics of the sample.

		Total sample (N = 214)	NC group (N = 32)	CI group (N = 182)
Demographics	Age^‡^	47.48 (7.35)	53.03 (6.31)	46.41 (7.77)
	Sex–women^§^	182 (85.04)	28 (87.50)	154 (84.61)
	Years of education^‡^	16.36 (5.89)	16.12 (2.75)	16.30 (3.35)
	Cognitive reserve^‡^	16.27 (3.06)	14.44 (2.86)	16.15 (5.89)
COVID-19 related characteristics	Time from diagnosis to assessment^‡^	508 (150.48)	518.56 (140.60)	505.98 (150.48)
	COVID-19 symptoms^§^	206 (96.26)	31 (96.87)	175 (96.15)
	COVID-19 diagnosis^§^	185 (86.44)	28 (87.50)	157 (86.26)
	Post COVID-19 syndrome diagnosis^§^	179 (83.64)	23 (7.80)	156 (85.71)
	Hospitalization^§^	51 (23.7)	7 (21.8)	44 (24.04)
	Days of hospitalization (range)	1–180	3–40	1–180
	ICU^‡^	6 (2.7)	2 (6.25)	4 (1.58)
	Headache^§^	173 (80.84)	24 (75)	149 (81.86)
	Anosmia^§^	55 (25.70)	7 (21.87)	48 (26.37)
	Ageusia^§^	56 (26.04)	6 (18.75)	50 (27.32)
	Vaccine^§^	188 (87.04)	29 (90.62)	159 (86.88)
Cognitive complaints after infection	Attention/concentration^§^	188 (87.85)	26 (81.25)	162 (89.01)
	Speed processing^§^	163 (76.16)	21 (65.62)	142 (78.02)
	Anomia^§^	185 (86.44)	27 (84.37)	158 (86.81)
	Short-term memory^§^	163 (76.16)	21 (65.62)	142 (78.57)
	Long-term memory^§^	103 (47.90)	11 (34.37)	92 (50.27)
	Reading difficulties^§^	149 (69.62)	22 (68.75)	127 (69.78)
	Multitasking^§^	161 (75.23)	24 (75)	137 (75.27)

^†^NC group = normal cognition group (patients with normal scores in all cognitive tests), CI group = cognitive impairment group (patients with at least one test with impaired scores).

^‡^Scores in terms of mean (SD), ^§^scores in terms of N (percentage of patients).

The eligibility criteria for participation in the study were: to be over 18 years of age, to have a confirmed diagnosis of COVID-19 or RT-PCR positive (or other diagnostic test) of at least 4 months prior to participation in the study, to have symptoms suggestive of post COVID-19 syndrome and, either be in the process of diagnostic confirmation or already have a confirmed diagnosis, to present neurological complaints that appeared during or after Sars-Cov-2 infection, not to suffer from brain damage, neurodegenerative, autoimmune or psychiatric disease that could lead to cognitive problems, and no history of alcohol or drug abuse.

Before undergoing the neuropsychological assessment, patients first completed a questionnaire providing information about their sociodemographic data, medical history, and clinical and cognitive complaints related to post COVID-19 syndrome symptoms. In addition, they also fulfilled a cognitive reserve questionnaire^16^.

The study is in accordance with the World Medical Association Declaration of Helsinki and has been approved by the ethics committee of Principality of Asturias (CEImPA code 2021.487). All patients were informed of the purpose of the study, as well as the possibility of dropping out at any time, and signed an informed consent prior to the neuropsychological evaluation.

### Neuropsychological assessment protocol and procedure

The neuropsychological protocol included the following tests: Brief Test of Attention (BTA)^17^; digits span forward and backward; TAVEC^18^) and all measures were included: TAVEC A (learning phase), TAVEC B (interference list), TAVEC FIR (free inmediate recall), TAVEC CIR (cued inmediate recall), TAVEC FLR (free long recall), TAVEC CLR (cued long recall) TAVEC rec (recognition); Rey-Osterrieth Complex Figure (ROCF) (copy and recall at 3 and 30 min^19^); Stroop Color-Word Interference test^20^; WAIS-IV Matrix reasoning^21^; fluency tasks: semantic (animals), phonological (words beginning with “p”), excluded (words without “e”) and actions and an object naming task from BETA test^22^.

Due to the pandemic situation and the restrictions imposed by the government, the neuropsychological evaluations were conducted online by trained neuropsychologists using Zoom or Microsoft Teams. For this reason, mostly oral tests were selected. For the non-verbal tests, such as the ROCF, Stroop, the WAIS-IV Matrix reasoning and the BETA subtest, a powerpoint presentation was prepared with each of the tests scanned in high quality. Given that the Stroop Color-Word Interference test is normally used in paper format and that on this particular occasion we had to adapt it for telematic use, a group of 50 healthy controls performed its three subtests under the same conditions as the patients. All the controls had normal scores on this test, so the results found in our sample, in our opinion, cannot be attributable to the type of assessment performed.

The raw scores obtained by every patient in each test were converted into standarized scores. In the case of TAVEC and BETA, Z-scores were used and for the rest of the tests scalar scores were available for Spanish population^23^. Z-Scores between − 1.5 and − 2.99 (corresponding to scalar scores between 4 and 6) were considered *mild impaired* and scores under -3 SD (corresponding to scalar scores ≤ 3) were considered *severe impaired* for this purpose.

To analyze the differences according to the age of the patients, we considered a group of participants between 26 and 39 years old (N = 29); a second group between 40 and 49 years old (N = 97) and a third group between 50 and 64 years old (N = 88).

Given the characteristics of the sample and the type of variables, nonparametric statistics analyses were performed (Spearman, Kruskall-Wallis and U-Mann Whitney). The effect size was estimated with Hedges correction from Cohen´s to nonparametric analyses.

## Results

Among the most common cognitive complaints reported by patients were difficulties in concentration (87.85%), anomia or word-finding difficulties during speech (86.44%), speed of processing and remembering recent events (76.16%), multitasking (75.23%) and reading aloud or reading comprehension (69.62%).

A correlation analysis was conducted in order to find out whether some of the sociodemographic variables (age, years of education, cognitive reserve or time of disease) were related to neuropsychological test performance. The results showed that age correlates with almost all neuropsychological tests, as can be seen in Table 2. All the correlations found between the neuropsychological tests and age were positive (i.e. the older the age, the better the scores on the tests), except in the ROCF copying task, in which a negative correlation was found. The same occurs with the relationship between cognitive reserve and years of education, all correlations were positive. In the case of duration of illness, a significant and positive correlation was found with TAVEC-A and the ROCF IR, implying that the longer the disease course, the better the scores on these tests.

**Table 2:** Correlations of cognitive performance with sociodemographic and disease duration.

	Age	Cognitive reserve	Years of school	Disease duration
BTA (brief test of attention)	0.19**	0.08	0.09	− 0.07
Digits forward	0.11	0.13	0.03	0.01
Digits backward	0.26**	0.10	0.03	0.01
TAVEC-A (learning phase)	0.19**	0.23**	0.25**	0.13*
TAVEC-B (interference list)	0.16*	0.12	0.03	0.01
TAVEC-FIR (free immediate recall)	0.22*	0.21**	0.18**	0.04
TAVEC-CIR (cued immediate recall)	0.12	0.17*	0.20**	0.02
TAVEC-FLR (free delayed recall)	0.16*	0.17*	0.15*	0.02
TAVEC-CLR (cued delayed recall)	0.21*	0.10	0.18*	0.04
TAVEC-rec (recognition phase)	0.14*	0.19*	0.17*	0.03
ROCF IR (immediate recall)	0.11	0.13	0.08	0.15*
ROCF LR (delayed recall)	0.11	0.11	0.04	0.10
ROCF copy	− 0.14*	0.07	0.04	− 0.02
ROCF copy—time	0.19*	0.01	0.07	0.10
Stroop word reading	0.30*	0.09	0.10	0.05
Stroop color naming	0.16*	0.10	0.09	− 0.02
Stroop word-color interference	0.27**	0.10	0.12	0.04
Matrix reasoning	0.33**	0.20**	0.10	0.01
Phonological fluency	0.14*	0.16*	0.03	− 0.01
Exclude fluency	0.18**	0.13	0.06	− 0.03
Semantic fluency	0.29**	0.15*	0.07	− 0.07
Object naming	0.08	− 0.01	0.04	− 0.12

*p < *0.05*, **p < *0.01*.

### Hospitalized vs. non-hospitalized patients

The findings in the neuropsychological assessments were compared between the group of patients who had required hospitalization (N = 51) and the group of patients who had not been hospitalized (N = 163). In none of the tests used any statistically significant differences were found between both groups. Significant differences were found between these two groups only for the variable age (U = 2922; p < 0.05), since the patients who required hospitalization were significantly older (mean age 50.09) than the other group (mean age 46.56).

### Cognitively intact vs. at least one impaired test patients

A total of 14.88% participants exhibited normal cognitive performance in all the neuropsychological tests. This group of participants had a mean age of 53.03 (SD = 6.31). When we compared this group with the group of patients showing impaired scores in some test, we found that the individuals without any deficiency score were, again, significantly older (U = 1492; p < 0.01). We also found differences in the cognitive reserve score, being the cognitively intact group the one with the lower cognitive reserve (U = 1706; p < 0.01), which rules out that their better performance is due to a higher cognitive reserve. There were no differences between the two groups in years of education or time of evolution.

With respect to the neuropsychological testing outcomes, as would be expected, there were statistically significant differences between the two groups in all tests used except for the ROCF copy (U = 2254; p > 0.05), ROCF copy-time (U = 2405; p > 0.05) and object naming (U = 2222; p > 0.05).

According to this, the results showed that 85.12% of the participants had an impaired score in at least one test. The test in which the greatest percentage of patients had mild impairment were Stroop W (38.90%), action fluency (32.80%), Stroop C (29%), Stroop W–C (28.40%) and TAVEC-FLR (25.10%). The tasks with the largest number of patients showing a severe impairment were the BTA (29.30%), Stroop W (20.90%), Stroop C (18.10%) and Stroop W–C (12.5%).

When the participants were analyzed by age-cohorts, it was observed that among the younger patients (ranged 26 to 39 years), 97.37% had at least one impaired score; in group 2 (ranged 40 to 49 years), the percentage was 91.43% and in group 3 (ranged 50 to 64 years) was 71.1%.

### Comparisons between group 1 (26–39 age) and group 2 (40–49 age)

Statistical analyses revealed a better performance of group 2 and differences between both groups in TAVEC A and TAVEC B, and the effect size can be considered as moderate (0.5 and 0.41, respectively). In the remaining neuropsychological tasks, no statistically significant differences were found. These results show that younger patients have more difficulties in learning verbal information.

### Comparisons between group 2 (40–49 age) and group 3 (50–64 age)

Statistical analyses showed a superior performance of group 3 and an effect size greater than 0.5 in the Stroop WC, Stroop W, ROCF copy time. In the remaining tasks in which statistically significant differences were found (Stroop C, ROCF LR, backward digits and matrixes), the effects were small. These results suggest that group 2 performed worse in tasks assessing processing speed, executive functions and visual memory.

### Comparisons between group 1 (26–39 age) and group 3 (50–64 age)

The results obtained by group 3 are significantly higher in all neuropsychological tasks in which statistically significant differences were observed. In addition, a large effect size (> 0.8) is reached in semantic fluency, backward digits, and Stroop W, while a moderate effect size in Stroop W–C, BTA and TAVEC FIR was found. This performance of younger patients suggests that they have more difficulties than older patients in at least one test of all cognitive domains assessed (language, executive functions, processing speed, attention, and verbal short-term memory).

Table 3 contains the results in all neuropsychological tests with standardized scores, the tests in which there are differences among the three groups analyzed and the effect size for each test.

**Table 3:** Results in all neuropsychological test by age-groups.

Cognitive domain	Test	Standarized scores mean (SD)											
		Group 1	Group 2	Group 3	Group 1 vs. 2			Group 2 vs. Group 3			Group 1 vs. Group 3		
					Kruskal Wallis	p value	effect size	Kruskal Wallis	p value	Effect size	Kruskal Wallis	p value	Effect size
Attention	BTA	14.79 (3.80)	16.52 (6.69)	16.18 (3.28)	− 24.35	0.18	0.39	− 10.09	0.79	0.16	− 34.45	0.02	0.58
	Digit forward	8.10 (3.48)	8.41 (3.16)	8.67 (2.69)	2.28	0.32	0.09	2.28	0.32	0.08	2.28	0.32	0.19
Working memory	Digit backward	7.79 (2.45)	9.15 (2.82)	10.15 (2.79)	− 25.37	0.15	0.49	− 31.85	< 0.001	0.35	− 57.22	< 0.001	0.86
Verbal memory	TAVEC A	− 1.05 (1.34)	− 0.44 (1.17)	− 0.44 (1.05)	− 31.36	0.04	0.5	1.89	1	0.03	− 29.44	0.07	0.53
	TAVEC B	− 0.86 (1.20)	− 0.41 (1.03)	− 0.43 (0.98)	− 32.67	0.03	0.41	4.7	1	0.01	− 27.96	0.11	0.39
	TAVEC FIR	− 0.92 (1.27)	− 0.31 (1.10)	− 0.25 (1.22)	− 30.44	0.06	0.53	− 4.41	1	0.04	− 34.86	0.02	0.53
	TAVEC CIR	− 0.51 (1.33)	− 0.18 (1.10)	− 0.19 (1.30)	1.85	0.39	0.28	1.85	0.39	0.01	1.85	0.39	0.24
	TAVEC FLR	− 1.18 (1.73)	− 0.66 (1.28)	− 0.61 (1.47)	2.30	0.31	0.37	2.30	0.31	0.04	2.30	0.31	0.37
	TAVEC CLR	− 1.11 (1.67)	− 0.50 (1.25)	− 0.40 (1.42)	5.12	0.07	0.44	5.12	0.07	0.07	5.12	0.07	0.47
	TAVEC rec	− 0.82 (2.07)	− 0.53 (1.26)	− 0.41 (1.28)	1.11	0.57	0.19	1.11	0.57	0.09	1.11	0.57	0.26
Visual memory	FCR IR	9.04 (2.88)	9.24 (3.24)	10 (2.48)	2.57	0.27	0.06	2.57	0.27	0.26	2.57	0.27	0.37
	FCR LR	9.39 (2.65)	8.83 (3.13)	10.10 (2.51)	11.99	1.00	0.18	− 25.98	0.01	0.44	− 13.93	0.87	0.27
Visuoespatial/executive function	FCR copy	11.41 (5.20)	10.42 (3.51)	9.99 (3.13)	1.66	0.43	0.24	1.66	0.43	0.12	1.66	0.43	0.37
	FCR copy—time	10.46 (3.04)	10.22 (3.12)	12.02 (2.72)	8.01	1.00	0.07	− 38.41	< 0.001	0.61	− 30.41	0.06	0.57
Speed processing	Stroop W	4.68 (3.36)	5.23 (3.53)	7.26 (3.12)	− 9.75	1.00	0.15	− 39.46	< 0.001	0.62	− 49.22	< 0.001	0.8
	Stroop C	6.54 (3.45)	5.98 (3.65)	7.62 (3.11)	10.81	1.00	0.15	− 28.81	< 0.005	0.47	− 18	0.51	0.33
Executive function	Stroop WC	6.46 (3.59)	6.29 (3.48)	8.82 (2.88)	3.16	1.00	0.04	− 44.5	< 0.001	0.78	− 41.33	< 0.005	0.76
	Matrix	7.45 (2.98)	8.99 (2.92)	9.82 (3.10)	− 27.33	0.10	0.52	− 45.69	< 0.001	0.27	− 18.29	0.12	0.76
	Phonological F	8.45 (3.75)	9.63 (3.13)	9.51 (2.47)	1.62	0.44	0.37	1.62	0.44	0.04	1.62	0.44	0.37
	Exclude F	9.24 (3.13)	10.05 (3.08)	10.77 (2.90)	4.90	0.08	0.26	4.90	0.08	0.23	4.90	0.08	0.51
Language	Semantic F	7.14 (3.31)	8.72 (3.43)	9.73 (2.71)	− 24.67	0.17	0.46	− 18.48	0.12	0.29	− 43.15	< 0.005	0.86
	Actions F	8.03 (2.97)	8.23 (3.19)	*	2.06	3.55	0.06	2.06	0.06	*	2.06	3.55	*
	Object naming	28.29 (1.82)	28.66 (1.24)	28.80 (1.12)	1.08	0.58	0.26	1.08	0.58	0.12	1.08	0.58	0.38

### Percentage of patients with mild and severe deficit by age groups considering those percentages above 25% of patients impaired

#### Group 1

In the group of younger participants, the tasks in which most patients showed a mild impairment were: TAVEC-A (44.8%), TAVEC-FIR (41. 40%), Stroop W (31.30%), action fluency (31%), ROCF copy (29.60%), Stroop C (28.60%), Stroop W–C (28.60%), matrix reasoning (27.60%) and TAVEC-CIR (27.60%). On the other hand, the tasks in which the highest proportion of patients exhibited a severe deficit were the BTA (41.40%), Stroop W (35.7%), Stroop W–C (25%).

#### Group 2

In this group, the patients exhibited moderate impairment in Stroop W (39.20%), Stroop W–C (34.70%), action fluency (33.30%), Stroop C (29.90%), semantic fluency (28.10%) and TAVEC-FLR (27.80%). The tasks in which the highest proportion of patients showed a moderate deficit were the BTA (30.90%), Stroop W (30.90%) and Stroop C (27.08%). The remaining tasks were all below 20% of patients with moderate deficits.

#### Group 3

In the oldest group of participants, the task in which there was a higher proportion of patients with mild impaired scores was the Stroop W (32.35%), and the only task in which there were more than 10% of patients with moderate deficits was the BTA (22.80%).

A more comprehensive description of the percentage of patients in each age group with scores above the mean, at the mean, with mild deficits and with moderate deficits can be found in Table 4.

**Table 4:** Percentage of patients with standardized scores in the mean, above the mean (Z > 1.5), with mild impairment (Z < − 1.5) and severe impairment (Z < − 3).

Cognitive domain	Test	% Z > 1.5				Mean				% Z < − 1.5				%Z < − 3			
		Total	Group 1	Group 2	Group 3	Total	Group 1	Group 2	Group 3	Total	Group 1	Group 2	Group 3	Total	Group 1	Group 2	Group 3
Attention	BTA	44.20	27.60	42.30	55.30	20.00	24.10	21.60	13.85	6.50	6.90	5.20	4.45	29.30	41.40	30.90	26.40
	Digit forward	3.70	3.40	5.20	10.65	75.80	72.40	74.20	83.60	20.00	20.70	20.60	10.75	0.50	3.40	0.00	0.00
Working memory	Digit backward	3.70	0.00	4.10	6.90	88.80	82.80	90.70	89.30	6.50	17.20	4.10	3.15	0.90	0.00	1.00	1.30
Verbal memory	TAVEC A	2.80	0.00	2.50	6.25	75.30	51.70	78.40	79.95	20.50	44.80	16.50	13.50	1.40	3.40	2.10	0.00
	TAVEC B	4.70	6.90	3.80	6.90	77.70	69.00	79.40	79.25	16.70	24.10	14.40	8.50	0.90	0.00	2.10	0.00
	TAVEC FIR	4.20	0.00	6.30	13.15	73.50	58.60	79.40	75.45	22.30	41.40	18.60	8.85	0.00	0.00	0.00	0.00
	TAVEC CIR	4.20	3.40	6.30	3.15	80.00	69.00	83.50	88.60	14.40	27.60	13.40	11.40	1.40	0.00	0.00	1.90
	TAVEC FLR	3.30	3.40	5.10	7.55	64.20	51.70	67.00	71.65	25.10	24.10	27.80	11.35	7.40	20.70	4.10	3.80
	TAVEC CLR	3.30	3.40	5.10	2.55	68.80	58.60	72.20	82.30	21.40	17.20	21.60	12.05	6.50	20.70	4.10	2.55
	TAVEC rec	0.00	0.00	0.00	0.00	76.70	65.50	79.40	82.95	17.70	20.70	18.60	17.65	5.60	13.80	2.10	3.80
Visual memory	FCR IR	2.80	0.00	4.20	10.65	82.90	85.20	75.80	81.20	11.40	11.10	15.80	18.25	2.40	0.00	4.20	1.30
	FCR LR	1.90	0.00	2.10	1.25	83.00	89.30	75.80	89.30	13.70	7.10	20.00	9.45	1.10	3.60	2.10	0.00
Visuoespatial/executive function	FCR copy	17.50	40.70	15.80	6.95	73.00	29.60	75.80	90.50	9.00	29.60	7.40	2.55	0.50	0.00	1.10	0.00
	FCR copy—time	13.30	3.60	11.70	14.50	80.10	89.30	78.70	83.60	6.20	7.10	9.60	1.25	0.50	0.00	0.00	1.40
Speed processing	Stroop W	0.90	0.00	0.00	1.30	39.30	25.00	29.90	63.80	38.90	31.30	39.20	32.35	20.90	35.70	30.90	5.10
	Stroop C	0.00	0.00	0.00	0.00	52.90	50.00	42.30	75.55	29.00	28.60	29.90	21.20	18.10	21.40	27.08	6.50
Executive functions	Stroop WC	1.90	0.00	2.10	1.30	57.20	46.40	43.20	81.40	28.40	28.60	34.70	16.05	12.50	25.00	20.00	0.00
	Matrix	4.20	0.00	5.20	6.90	75.20	69.00	74.00	79.25	20.10	27.60	20.80	8.85	0.50	3.40	0.00	0.00
	Phonological F	4.70	3.40	7.30	1.25	81.80	72.40	78.10	89.30	10.30	13.80	12.50	3.80	2.80	10.30	2.10	0.80
	Exclude F	7.90	3.40	6.30	15.05	83.20	75.90	85.40	81.75	7.00	17.20	6.30	2.55	1.90	3.40	2.10	0.00
Language	Semantic F	4.20	0.00	6.30	6.25	72.00	58.60	62.50	88.05	20.10	24.10	28.10	5.70	3.70	17.20	3.10	0.00
	Actions F	0.00	0.00	0.00	*	62.30	65.50	61.30	*	32.80	31.00	33.30	*	4.90	3.40	5.40	*
	Object naming	0.00	0.00	0.00	0.00	100.00	100.00	100.00	100.00	0.00	0.00	0.00	0.00	0.00	0.00	0.00	0.00

## Discussion

Given the scarcity of data on the cognitive characteristics of patients with post COVID-19 syndrome, most of them obtained from relatively small samples (we have not found any previous study with a sample of more than 70 patients) and with such a short time of evolution that it is impossible to know whether cognitive symptoms persist over time or disappear, the objective of this study was to assess, using a comprehensive neuropsychological protocol, a large sample of post COVID-19 syndrome patients, as well as to determine which characteristics could influence their performance. Thus, we evaluated a total of 214 patients who still had symptoms compatible with a diagnosis of post COVID-19 syndrome. The characteristics of the sample in terms of age and sex (mean 47.48 and 85.04% female) coincide with those found in previous studies^3,4,12^. The distribution in terms of biological sex could indirectly support one of the hypotheses that try to explain this disorder, specifically the autoimmune hypothesis, since several studies^24,25^ have found that this type of disease affects women more than men in a ratio of 80–20%. Liang’s study^25^ has even allowed the identification of an inflammatory pathway called ‘VGLL3’, which plays an essential role in the control of the female immune network especially, but also found in males with autoimmune diseases.

Regarding the results of the neuropsychological battery, it should be noted that more than 85% of the patients showed alterations in at least one neuropsychological test. This result is consistent with that found in the studies by Krishnan et al.^11^ and Delgado-Alonso et al.^12^, but contradicts the results of Dressing et al.^13^, who only observed mild cognitive impairment in one of the 31 participants in the study. This result could be explained by the characteristics of our sample, since in this research we only present the results of patients with post COVID-19 syndrome and who also experienced a mild severity of the disease, given that only 51 people needed hospitalization. Moreover, the statistical results reveal no differences in any of the tests between hospitalized and non-hospitalized patients, so it can be ruled out that the deficits found are a consequence of a severe disease and are in fact post-COVID sequelae.

More interestingly, both the presence and severity of impairments seem to have a relationship with age in the opposite direction to that expected, i.e. younger patients show impairment in some cognitive domain more frequently than older ones, and the severity of this impairment is greater in these younger patients. This result has been corroborated both by correlation analysis and by comparisons between groups of patients according to their age, and is consistent with that reported in Devita et al.’s study^26^. This outcome is a priori contrary to expectations, since younger age usually induces a better prognosis in virtually all disorders with cognitive deficits, such as dementia and acquired brain injury. However, it again seems to support the autoimmune hypothesis, since aging affects the immune system by weakening it, so that the autoimmune response will also be weaker.

Regarding the different cognitive functions, our results reveal that attention is the one that shows the greatest deficit, regardless of the age of the patients (more than 25% of patients in each age group exhibit a moderate attention deficit). With this in mind, it is not surprising that most of the studies assessing attention reviewed by Bertuccelli et al.^10^ show deficits, even though many of them used screening tests to carry out the evaluations.

Processing speed was also a cognitive process that was impaired relatively homogeneously across age groups, however, the results indicate that the youngest patients show a much slower processing speed (33.3% of those under 50 years of age) compared to the oldest (5.10% of those over 50 years of age). These results are similar to those reported in other studies^11,12^

The highest percentage of patients with severe verbal memory impairment was also found in the younger patients group. In the long-term memory, 20.7% of the youngest group showed a severe deficit and 24.1% a mild impairment, while a minimum proportion of the elderly showed alterations in this area. Some studies that used verbal memory tests found contradictory results. Thus, in the study by Di Pietro et al.^27^ only 2 out of 12 patients showed verbal memory difficulties, while Delgado-Alonso et al.^12^ identify verbal memory as one of the areas with the greatest deficits in their sample of 50 patients with post COVID-19 syndrome. The results observed in the verbal memory test might be related to the temporal hypometabolism, and more concretely to the hippocampal dysfunction found by Guedj et al.^14^, since the hippocampus is a crucial structure in the encoding of new information^28^.

Taken together the more semantic verbal fluency tasks (semantic fluency and actions fluency), the effect of age is clearly observed, since 29.12% of patients aged under 50 showed a mild deficit and 7.27% a moderate deficit, while only 5.70% of those aged over 50 showed a mild deficit and none a moderate deficit. In general, language is the most understudied cognitive function in most of the articles^10^, even less when considering the age of the participants, so these data are of special relevance and interest. Similarly as in the case of memory, the results obtained in semantic fluency can be understood as an alteration in the functioning of the temporal lobe^14^, since this region plays an important role in this particular kind of verbal fluency^29^.

In terms of executive functions, similar findings were observed for working memory, reasoning, inhibition and even lexical searching. There were differences between groups’ performance in working memory, being the youngest the ones with the highest percentage of patients with mild deficits (21.30% of those under 50 years of age) compared to those over 50 years of age (6.30%). Similar differences between age groups were found in the reasoning task. In this case, 24.2% of those under 50 showed a mild deficit and 3.4% a moderate deficit, while only 8.85% of those over 50 showed a mild deficit in this task. In the case of inhibition (assessed with the Stroop W–C), 31.65% of patients under 50 have a mild deficit and 22.5% a moderate deficit and only 16.05% of patients above 50 have a mild deficit while none of the patients had a moderate deficit. Phonological and excluded fluency also can be considered as an executive function since this lexical search activates frontal regions^30^. Taken together, 12.45% of patients under 50 years of age show a mild deficit and 4.5% a moderate deficit, only 3.17% of patients over 50 years of age show a mild deficit in this task. Considering all these cognitive domains as executive function, other studies also found worse performance in this sort of tests. Thus, in the study by Delgado-Alonso et al.^12^, executive functions were the cognitive domain in which their sample of participants had the most difficulties, while in the review study conducted by Bertuccelli^10^, impaired cognitive functions were found in half of the 16 studies that tested them.

Regarding the duration of the disease, understood as the time elapsed between the date of infection and the date of evaluation, the mean for all patients was 508 days. This suggests that cognitive difficulties are often maintained even up to 2 years or more. However, on some tasks, such as the verbal learning phase and immediate recall of visual memory, patients with longer evolution time showed better results, suggesting that some cognitive areas might experience some improvement over time.

Finally, although several studies^31–36^ point to the determinant role of cognitive reserve in the deterioration of abilities, it seems to have little impact on the cognitive performance of our patients, although a relationship was found with some tasks (the higher the cognitive reserve, the better the scores in the TAVEC-A, in the reasoning task, and in phonological and semantic verbal fluency). Previous research concerning the protective role of cognitive reserve have found that it has a significant influence on language, executive functions and memory^37^, which is consistent with our results.

Although one of the potential limitations of the study is the neuropsychological assessment was performed online, a recent review and other studies had demonstrated that telemedicine is a valid approach to perform neuropsychological assessments in adult patients^38,39^. Furthermore, according to the study by Matias-Guiu et al.^40^ in which our cohort of patients was compared with a similar cohort evaluated in person, there are no differences in the results of the two groups of patients.

In conclusion, the results presented here reveal that at least 85% of the participants exhibit deficits in one neuropsychological test. Also, the youngest patients were those who showed the most marked and heterogeneous cognitive impairment, while the oldest patients maintained their cognitive functions preserved to a greater extent with only a mild impairment in attention and speed processing. Precisely both functions were the cognitive processes that are most deficient and homogeneously identified across the different age subgroups.

## Data Availability

The data that support the findings of this study are available from the corresponding author, (gonzaleznmaria@uniovi.es) upon reasonable request.
